# Comparative analysis of outcomes in high KDPI spectrum kidney transplants using unsupervised machine learning algorithm

**DOI:** 10.1371/journal.pone.0324265

**Published:** 2025-08-26

**Authors:** Mahmoudreza Moein, Alireza Golkarieh, Isabella Vlassis, Reza Saidi, Michael Lioudis

**Affiliations:** 1 Division of Transplant Services, Department of Surgery, SUNY Upstate Medical University, Syracuse, New York, United States of America; 2 Department of Computer Science and Engineering, Oakland University, Rochester, Michigan, United States of America; 3 Division of Nephrology, Department of Medicine, SUNY Upstate Medical University, Syracuse, New York, United States of America; Keck Hospital of USC, UNITED STATES OF AMERICA

## Abstract

**Background:**

The Kidney Donor Profile Index (KDPI) is a continuous metric used to estimate the risk of allograft failure for kidneys from deceased donors. Lower KDPI scores are associated with longer post-transplant kidney function. This study aims to evaluate the outcomes of kidney transplantation using high-KDPI kidneys (98–100%) compared to those with moderately high KDPI scores (85–97%), employing a novel case-matching approach using machine learning.

**Methods:**

We conducted a retrospective analysis of the United Network for Organ Sharing (UNOS) database, examining kidney transplants performed in the United States between January 2000 and May 2020. An unsupervised machine learning algorithm was used to match recipients of KDPI 98–100% kidneys with recipients of KDPI 85–97% kidneys based on key baseline characteristics, including recipient age, body mass index (BMI), cold ischemia time, HLA mismatch, ethnicity, and gender.

**Results:**

A total of 6,624 matched cases were selected for analysis. The mean follow-up duration was 4.5 years for the KDPI 98–100% cohort and 4.6 years for the KDPI 85–97% cohort. The five-year allograft survival was 51.7% for the KDPI 98–100% group versus 58% for the KDPI 85–97% group (P < 0.001). Asian recipients showed the highest survival in both cohorts (68% vs. 69%). Donation after circulatory death (DCD) status did not significantly impact outcomes. Across the full cohort, 1,819 cases of allograft failure were recorded, with chronic rejection being the leading cause (28.4% vs. 30%, P = 0.56).

**Conclusion:**

Transplantation with high-KDPI kidneys, though associated with lower survival rates, remains a viable option for expanding the donor pool. With appropriate recipient selection, high-KDPI kidneys can improve patient quality of life, reduce wait times, and lower healthcare costs. Our findings support a more nuanced approach to organ allocation using advanced matching strategies.

## Introduction

Kidney transplantation (KT) for patients diagnosed with end stage renal disease (ESRD) provides the best opportunity to improve the quality of life. When compared to dialysis, KT is attributed to lower long-term morbidity and mortality as well as improved cost efficiency [1–3]. In the past, there has been a prevalent imbalance between the number of patients on organ recipient waiting lists and the number of available donor organs. Over the last decade, new strategies have been successfully implemented to remediate the disparity. The United States Renal Data System (USRDS) reported an overall increase in the deceased donor kidney transplant rate, rising from 2.3 per 100 person-years in 2013 to 3.3 per 100 person-years in 2022 [4]. According to the most recent Organ Procurement and Transplantation Network (OPTN) data, there was an 8.1% increase in kidney transplants from 2020 to 2021. In addition, 2021 had a record number of kidney transplants which are attributed to the growth in deceased donor kidney transplants. Deceased donor kidney transplant (DDKT) rates among waitlisted candidates continued to increase in 2021. Among DDKTs, an 80.7% 5-year allograft survival was noted for recipients aged 18–34 as well as a 68% 5-year allograft survival for recipients aged 65 years and older [5].

In the past, kidney allocation systems designated a donor kidney as either a standard criteria donor (SCD) or expanded criteria donor (ECD). Within these two categories, there was a wide variation in expected kidney function. Kidney Donor Profile Index (KDPI) provides more granularity as a calculated percentage intended to divide deceased donor kidneys into broad categories. KDPI is converted from the Kidney Donor Risk Index (KDRI), a continuous grading scale that reflects the risk of allograft failure with a transplanted kidney from a particular donor [6]. Utilizing ten donor variables, able to be measured prior to procurement, a linear percentage is calculated which summarizes the likelihood of allograft failure relative to reference donors from the previous year [7]. Lower KDPI scores are associated with longer estimated function; scores of 0% have the longest anticipated survival rate and 100% have the shortest anticipated survival rate [8].

Sixty-five percent of deceased donor kidneys have a KDPI between 21 and 85%. Kidneys that fall into this category are expected to function for around 9 years. A donor kidney with a KDPI exceeding 85% is expected to function for approximately five and a half years [8]. According to the OPTN/SRTR 2021 Annual Data Report: Kidney, five-year DDKT allograft survival was 64.0% for allografts with KDPI of 85% or greater.

In 2021, 24.6% of deceased donor kidneys recovered were not used for transplantation (discard rate), an all-time high. Greater discard rate was noted in deceased donor kidneys with KDPI >85% [5]. According to Husain et al., in a cohort study analyzing 280,041 wait-listed kidney transplant candidates in the US, 30% of candidates whom a deceased donor offer was declined on their behalf were eventually removed from the waiting-list before receiving an allograft or expired [9]. Reducing the unnecessary discard of deceased donor kidneys would maximize the utilization of a scarce resource. In addition, certain patient characteristics may not require a high-longevity kidney in which case accepting a kidney with a KDPI>85% would expand the opportunity for transplantation in a timely manner.

The aim of our study is to explore the difference in outcome of deceased donor kidney transplantation when using donor kidneys with a KDPI of 98% or above compared to donor kidneys with a KDPI 85–97%.

## Method and materials

### Study design

The Organ Procurement and Transplantation Network (OPTN) database was used to perform a retrospective registry analysis on Kidney Transplantation performed in the United States between January 1, 2000, and May 31, 2020. The study was IRB exempted and was in accordance with the STROBE Guidelines. The need for consent was waived by the ethics committee, as all data were fully anonymized before our access, by the OPTN.

Only KTs that include outcome data were included in this study. The study population was divided into two subgroups primarily based on the donor kidney KDPI, patients who received a kidney with a KDPI between 85%−97%, and recipient who received a kidney with a KDPI 98% to 100%. This study aimed to closely match the KDPI 98–100% cohort to the KDPI 85–97% cohort based on baseline characteristics known to be associated with lower allograft survival and overall survival, minimizing the influence of potential confounding factors [10–15]. Characteristic variables that were matched between the cohorts were recipients BMI, Age, cold ischemia time, HLA mismatch, recipients’ ethnicity, and recipients’ gender. Recipients who passed away within 30 days of KT, failed to appear at follow-up appointments, or were <18 years of age were excluded. Survival outcomes and allograft success rates were measured and compared between the patients in both time frames and subgroups at 5-years post-transplant. Various recipient characteristics were noted, such as age, gender, race, body mass index (BMI), and primary diagnosis. The recorded donor characteristics include age, gender, race, and cold ischemia time. Based on the OPTN definitions, kidney allograft failure had occurred when a recipient’s transplanted organ is removed, A recipient dies, or A recipient is placed on a chronic allograft support system. Acute allograft rejection is defined as a record of acute or hyperacute rejection, or a record on the OPTN Transplant Recipient Registration or Transplant Recipient Follow‐up Form of an anti‐rejection drug being administered. Only the first rejection event is counted, and it was biopsy-proven.

### Matching process

Unsupervised clustering algorithms are one of the most effective and common types of algorithms to find similar data points without the need for data labeling. This study aimed to closely match the KDPI 98–100% cohort to the KDPI 85–97% cohort based on baseline characteristics known to be associated with lower allograft survival and overall survival to be able to minimize the influence of potential confounding factors, [10–15]. As studies shows, these characteristic variables are: recipients BMI, Age, cold ischemia time, HLA mismatch, recipients’ ethnicity, and recipients’ gender, therefore they were the selected variables to match cases between the cohorts.

To ensure a balanced distribution of the selected confounding variables between the two cohorts, matching methods were employed to construct two cohorts—subsets of the KDPI 85–97% and KDPI 98–100% cohorts—based on the similarities of individuals between these two cohorts. Matching techniques enabled the assignment of the most similar case from the KDPI 85–97% cohort to each case in the KDPI 98–100% cohort, effectively balancing baseline characteristics in this non-randomized study.

When selecting the appropriate matching algorithm, two primary criteria were considered:

- Maximizing the number of matched cases
- Ensuring the treatment (KDPI 98%−100%) and control (KDPI 85%−97%) groups were as similar as possible.

Both propensity score matching and an unsupervised machine learning matching method, unsupervised clustering, were applied and compared in terms of the number of matched cases and the quality of the matches. As part of the data preprocessing, patients with missing values for the above features were excluded, and only cases with complete data were included and reported. The quality of the matches was assessed using sensitivity analysis including p-value comparisons, standard mean difference, and the evaluation of baseline characteristics of selected variables. Based on the results, the best matching of two groups in this dataset was achieved by unsupervised clustering algorithm with Manhattan distance which is the optimum solution for having both highest number of matched cases without replacement (duplicates), highest similarities of chosen variables (lowest Manhattan distance), and similar covariates distributions. Table 1 presents a comparison of match quality between the propensity score matching and unsupervised clustering matching methods.

**Table 1:** Comparison of data matching between study cohorts based on baseline characteristics using propensity score matching and unsupervised machine learning methods.

	Clustering with Manhattan Distance	Clustering with Cosine Distance	Propensity Score with Replacement	Propensity Score without Replacement
Number of selected cases from KDPI 98–100 group	1104	1102	944	1305
Number of selected cases from KDPI 85–97 group	5520	5510	4720	6505
**P-Values**	Clustering with Manhattan Distance	Clustering with Cosine Distance	Propensity Score with Replacement	Propensity Score without Replacement
Recipients’ BMI	0.99	0.85	0.85	0.86
Recipients’ age	0.45	0.41	0.93	0.94
Cold ischemia time	0.97	0.90	0.55	0.77
HLA mismatch	0.99	0.99	0.57	0.02
Recipients’ ethnicity	0.99	0.93	0.00	0.00
Recipients’ gender	1.0	1.0	0.7	0.05

Therefore, the unsupervised clustering algorithm was used to pick the most similar data point (case) from the KDPI 85–97% cohort to the data point (cases) from the KDPI 98–100% cohort. The clustering algorithm considered each data point in the KDPI 98–100% cohort as the centroid of a cluster (center of an independent cluster). Therefore, it generated 1301 clusters at the beginning because there are 1301 cases in the KDPI 98%−100% cohort, and then assigned each data point from the KDPI 85–97% cohort to the cluster with the most similar centroid, (data point from the KDPI 98–100% cohort), from those 1301 initiated clusters. Manhattan distance was considered as the metric to measure the similarity between the KDPI 85–97% cohort data points and the KDPI 98–100% cohort data points (centroids), so the most similar centroids for each assigning data point from the KDPI 85–97% is the centroid that has the smallest Manhattan distance from the assigning data point. After clustering all the KDPI 85–97% cohort data points, the clusters with more than 5 members were picked, 1104 clusters were chosen, and sorted the members of those clusters based on their distance from the centroid of the clusters that they belonged to. Then, the five closest data points to the centroid of each cluster (lowest Manhattan distance to the centroids) were chosen as the similar subset of the KDPI 85–97% cohort cases. Clusters with less than 5 assigned data points from the KDPI 85–97% cohort, were excluded as they did not have the minimum number of similar data points from the primary data. The missing value rate was less than 1%. During data cleaning and preprocessing, data points with missing values, partially missing values, or erroneous entries were excluded from the analysis.

**Fig 1:**
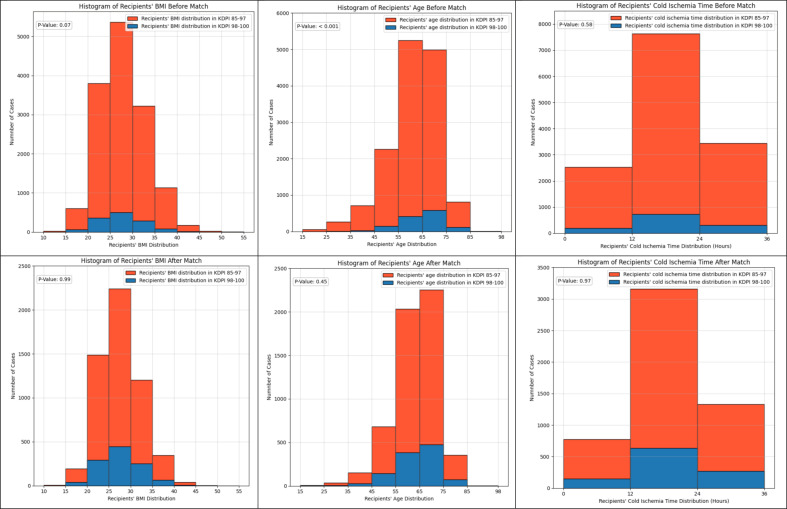
Baseline characteristics of recipients’ comparison before and after data match by the unsupervised machine learning algorithm.

Baseline characteristics of recipients was compared before and after data match by the unsupervised machine learning algorithm. (Fig 1)

### Outcomes definition

The primary objective was to assess the distinction in allograft success and survival rates among patients who received KTs based on the KDPI. Allograft failure was defined as either removal of the transplanted kidney, return to dialysis or re-registering for a kidney transplant. The reported allograft survival rates were all death censored. All eligible patients were followed until the criteria defined above were met or until the end of the study period.

### Statistical analysis

Python (Pandas, NumPy, Sklearn, and Matplotlib) was utilized, for the purpose of machine learning algorithm design and implementation. The analysis of baseline characteristics and outcomes of each study group was done using IBM SPSS 29 and STATA 18 software. Multiple tests comprised our analysis depending on the type of variable that was measured: T-test for univariate analysis of continuous variables, chi-square for categorical variables, and Kaplan-Meier curves for patient and allograft survival rates. Associated with the continuous variables, mean and standard deviations were calculated, while proportions and percentages were calculated for categorical variables. To determine the influence of multivariate factors on allograft survival, a cox regression analysis was conducted to examine recipient gender, ethnicity, BMI, and age, as well as donors’ baseline characteristic factors. All P-values greater than 0.05 were considered significant, and values less than 0.1 were considered a trend.

## Results

Following the unsupervised machine learning matching process, 6,624 cases were selected for inclusion in the study from a total of 15,727 primary cases identified after applying exclusion criteria (1,307 cases from the KDPI 98–100% cohort and 14,420 cases from the KDPI 85–97% cohort). 1104 cases from the KDPI 98–100% cohort were matched to 5520 cases from the KDPI 85–97% cohort, with a 1:5 match ratio. Cases that were selected by the algorithm were matched based on the recipients BMI, Age, cold ischemia time, HLA mismatch, recipients’ ethnicity, and recipients’ gender. The mean follow-up time was 4.5 years for the KDPI 98–100% cohort, and 4 years and 7 months for the KDPI 98–100% cohort. The demographic characteristics of both recipients and donors in each cohort stratified by KDPI are shown in Table 2. Donors’ age was slightly higher in the KDPI 98–100 cohort, as expected, and more kidneys were allocated from African American donors. Also, more kidneys were allocated for transplantation from DCD donors in the KDPI 85–97% cohort (11.6% vs. 4.8%, P < 0.001). Other than these, there was no significant difference between the two cohorts, in terms of baseline characteristics.

**Table 2:** Kidney transplant recipients’ and donors’ demographic characteristics comparison between KDPI 98-100% vs. KDPI 85-97% cohorts.

Characteristics	KDPI 98-100% (n= 1104)	KDPI 85-97% (n=5520)	P-value
Recipients’ Characteristics			
Age (mean±SD)	64±8	61±10	<0.001
Ethnicity			
White	431 (39%)	2159 (39.1%)	0.99
Black	368 (33.4%)	1855 (33.6%)	
Hispanic	182 (16.5%)	864 (15.7%)	
Asian	97 (8.8%)	507 (9.2%)	
Others	26 (2.3%)	135 (2.4%)	
Primary diagnosis			
FSGS	24 (2.2%)	221 (4%)	0.11
PKD	78 (7%)	350 (6.3%)	
Hypertensive nephrosclerosis	296 (26.8%)	1376 (24.9%)	
T2DM	325 (29.5%)	1482 (26.9%)	
Others	381 (34.5%)	2091 (37.9%)	
Sex			
Male	701 (63.5%)	3505 (63.5%)	0.99
Female	403 (36.5%)	2015 (36.5%)	
On dialysis			
Yes	893 (80.9%)	4532 (82.1%)	0.32
No	211 (19.1%)	988 (17.9%)	
BMI (mean±SD)	27.50±4.77	27.76±4.80	0.39
Days on waitlist (mean±SD)	682±598	816±696	<0.001
HLA mismatch			
0	27 (2.5%)	171 (3.1%)	0.99
1	12 (1.1%)	72 (1.3%)	
2	23 (2.1%)	110 (2%)	
3	125 (11.3%)	629 (11.4%)	
4	294 (26.6%)	1435 (26%)	
5	359 (32.5%)	1784 (32.3%)	
6	264 (23.9%)	1319 (23.9%)	
Peak PRA (mean)	9.73	10.26	0.54
Serum Albumin at transplant (mean±SD, mg/dl)	3.92±0.55	3.94±0.57	0.59
Serum creatinine at transplant (mean±SD, mg/dl)	7.29±3.04	7.72±3.10	0.01
Serum creatinine at discharge (mean±SD, mg/dl)	3.85±2.60	4.32±2.83	<0.001
DGF			
Yes	332 (30.1%)	1833 (33.2%)	0.16
No	772 (69.9%)	3687 (66.8%)	
CMV status			
Positive	828 (75%)	3897 (70.6%)	0.002
Negative	232 (21%)	1435 (26%)	
Known	44 (4%)	188 (3.4%)	
Length of hospitalization (mean, days)	8	8	0.59
Donors’ Characteristics			
Age (mean±SD)	67±8	59±12	<0.001
Sex			
Male	428 (38.8%)	2489 (45.1%)	0.08
Female	676 (61.2%)	3031 (54.9%)	
Ethnicity			
White	424 (38.4%)	3064 (55.5%)	<0.001
Black	539 (48.9%)	1612 (29.2%)	
Hispanic	102 (9.2%)	563 (10.2%)	
Asian	30 (2.7%)	215 (3.9%)	
Others	9 (0.8%)	66 (1.2%)	
BMI (mean±SD)	28.36±6.99	28.54±7.29	0.12
Cold ischemic time (hours±SD)	20.23±8.52	20.10±8.76	0.78
Kidney on pump			
Yes	648 (58.7%)	2992 (54.2%)	0.16
No	361 (32.7%)	1982 (35.9%)	
Known	95 (8.6%)	546 (9.9%)	
DCD donor			
Yes	53 (4.8%)	640 (11.6%)	<0.001
No	1051 (95.2%)	4880 (88.4%)	
Last Serum creatinine (mean±SD, mg/dl)	1.38±1.08	1.26±1.21	0.22

DCD: Donation after circulatory death, DGF: Delayed graft function, FSGS: Focal segmental glomerulosclerosis, PKD: Polycystic kidney disease, PRA: Panel reactive antibody, T2DM: Type 2 diabetes mellitus.

**Fig 2:**
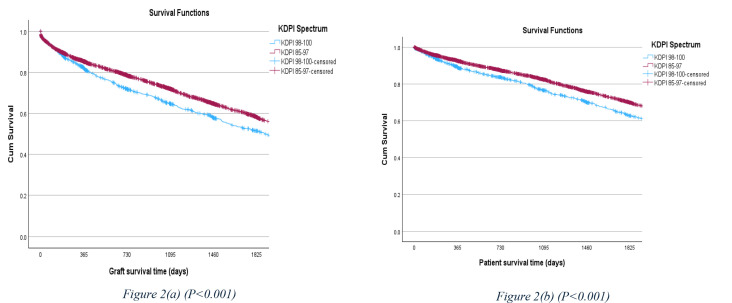
5-year allograft and patient survival rate comparison between the KDPI 98–100% and KDPI 85–97% cohorts.

Five-year allograft survival rate was calculated for each cohort. The overall five-year allograft survival rate was 51.7% for the KDPI 98–100% cohort, and 58% for the KDPI 85–97% cohort (P < 0.001). The five-year patient survival rate was 62.8% for the KDPI 98–100% cohort, and 69.8% for the KDPI 85–97% cohort (P < 0.001) (Fig 2).

Table 3 demonstrates the five-year allograft survival rate for each cohort, stratified by different factors. The allograft survival rate improved significantly in the 2011–2020 time frame, compared to 2000–2010 time frame, in both cohorts.

**Table 3:** The five-year allograft survival comparison between the KDPI 98-100% and KDPI 85-97% cohorts considering different factors.

Parameter	Five-year allograft survival KDPI 98-100	Five-year allograft survival KDPI 85-97	p value
Overall	51.7%	58%	<0.001
Transplant year			
2000-2010	46.7%	52.7%	<0.001
2011-2020	54.2%	59%	0.08
DGF			
Yes	39.2%	44%	0.003
No	55.2%	61.8%	<0.001
Ethnicity			
White	46.5%	54%	<0.001
Black	48%	54.6%	<0.001
Hispanic	53.4%	61.8%	0.056
Asian	68%	69%	0.87
DCD donor			
Yes	48.9%	56.4%	0.29
No	49.5%	56%	<0.001
Recipients’ age			
18-34	25.5%	44.7%	0.08
35-49	48.9%	58.2%	0.31
50-64	51%	58.8%	0.002
>65	48.9%	52.8%	0.005
On dialysis			
Yes	50%	53.3%	<0.01
No	50.1%	67.3%	<0.001

**Fig 3:**
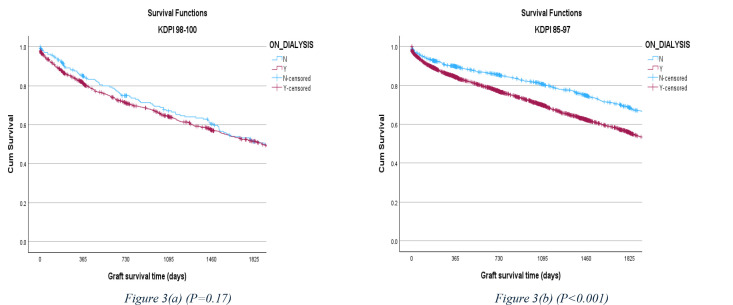
5-year allograft survival rate comparison, stratified by the history of maintenance dialysis between the KDPI 98–100% and KDPI 85–97% cohorts.

Interestingly, there was no significant difference between the recipients who were and were not on dialysis before the transplant in the KDPI 98–100% cohort, in terms of five-year allograft survival rate (50% vs. 50.1%, respectively). However, the five-year allograft survival rate difference was noticeable in the KDPI 85–97% cohort (53.3% vs. 67.3%, respectively), and Preemptive transplantation (transplantation without prior maintenance dialysis) is associated with longer allograft survival (Fig 3).

**Fig 4:**
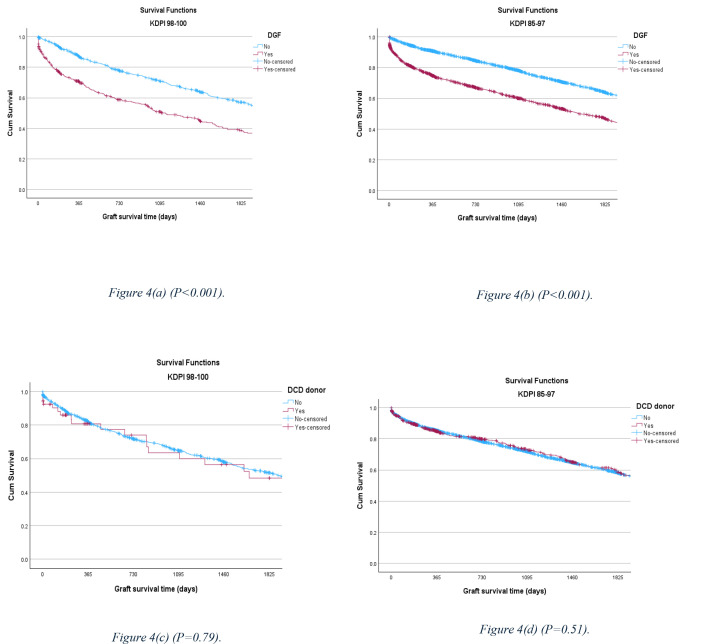
5-year allograft survival rate comparison, stratified by DGF and DCD between the KDPI 98–100% and KDPI 85–97% cohorts.

DGF is a strong predictor of long-term allograft outcome, and significantly decreases the five-year survival rate in both cohorts. [16,17] Also, receiving a kidney from a DCD donor did not significantly affect the outcome (Fig 4).

**Fig 5:**
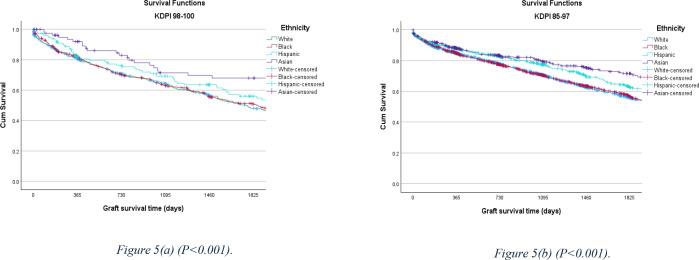
5-year allograft survival rate comparison, stratified by ethnicity between the KDPI 98–100% and KDPI 85–97% cohorts.

Recipients with Asian ethnicity showed a superior outcome in both cohorts with a 68% survival rate in the 98–100% KDPI cohort and 69% in the 85–97 KDPI% cohort (Fig 5).

**Fig 6:**
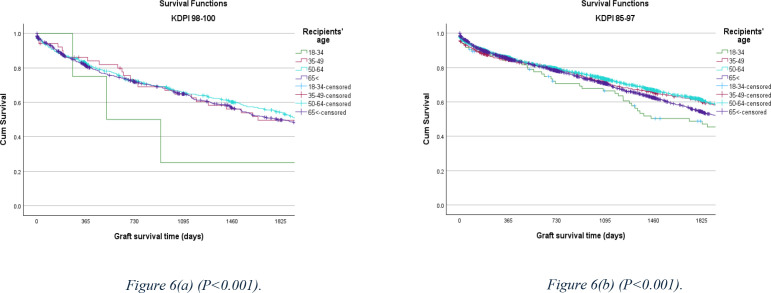
5-year allograft survival rate comparison, stratified by the recipients’ age between the KDPI 98–100% and KDPI 85–97% cohorts.

Among the different recipients’ age range, patients who were 35–64 years old and received a kidney with a KDPI 85–97% showed a better outcome (Fig 6).

Table 4 shows the comparison of the allograft failure etiology in KDPI 98–100% and KDPI 85–97% cohorts. A total of 1819 episodes of allograft failure were recorded and documented in the entire cohort. Chronic rejection was the main cause of allograft failure in both cohorts (28.4% in KDPI 98–100% vs. 30% in KDPI 85–97%, P = 0.56). None of the allograft failure causes were higher in the 98–100% KDPI cohort, compared to the KDPI 85–97% cohort.

**Table 4:** The etiology of allograft failure and comparison between the KDPI 98-100% and KDPI 85-97% cohorts.

Allograft failure causes (% total reported data)	Total cohort (n=1819)		OR with 95% CI	P-value
	KDPI 98-100% (n=332)	KDPI 85-97% (n=1487)		
Infection	18 (5.4 %)	75 (5%)	1.07 [0.63, 1.83]	0.77
Primary graft failure	32 (9.6%)	177 (11.9%)	0.78 [0.53, 1.17]	0.24
Recurrent disease	15 (4.5%)	67 (4.5%)	1.00 [0.56, 1.77]	0.99
Acute rejection	32 (9.6%)	186 (12.5%)	0.74 [0.50, 1.10]	0.14
Chronic rejection	94 (28.4%)	445 (30%)	0.92 [0.71, 1.20]	0.56
Vascular thrombosis	12 (3.6%)	53 (3.5%)	1.47 [0.77, 2.80]	0.23
BK (polyoma) virus	3 (0.9%)	30 (2%)	0.63 [0.19, 2.10]	0.63
Others	126 (38%)	454 (30.6%)	1.39 [1.08, 1.78]	0.008

## Discussion

Kidney transplantation is associated with lower long-term morbidity and mortality, as well as improved cost efficiency, for patients with end-stage renal disease who are eligible for transplant compared to those who remain on dialysis [1–3]. The KDPI scoring system allows measurement of the likelihood of allograft failure relative to reference donors from previous year. Longer estimated function and less likelihood of allograft failure are associated with lower KDPI scores. Sixty-five percent of deceased donor kidneys have a KDPI between 21 and 85% which have expected function around 9 years. The OPTN/SRTR 2021 Annual Data Report revealed that allografts with KDPI of 85% or greater had a 5-year DDKT allograft survival rate of 64.0%.

Thirty percent of candidates offered a deceased donor kidney which is declined on their behalf are removed from the waiting list before receiving an allograft or expire [9]. Greater discard of these kidneys was noted with KDPI >85% [5]. Crannell et al assessed the discard rate of deceased donor kidneys between 2012–2020 time frame, and they have found that kidneys with a KDPI above 85% are significantly discarded compared to lower KDPI kidneys, even compared to kidneys with a KDPI 83% or 84% and there was a lower utilization rate [18].

Our findings focus on the difference in outcome when utilizing donor kidneys with KDPI of 98% or above, compared to KDPI 85–97%. Utilization of high-KDPI kidneys increases access to transplantation, particularly for patients over the age of 60 or those who face challenges in finding a suitable donor match due to factors such as HLA sensitization, blood type incompatibility, or a history of prior kidney transplantation.

Vanin et al, conducted a similar study on their own patients who received high KDPI kidneys, in Colombia. They reported a 55.5% 2-year patient and allograft survival rate for their cohort. Contrary to their findings, the 2-year allograft survival rate in our study was over 70% in both cohorts and above 80% for the 2-year patient survival rate. A contributing factor to this difference could be the small sample size in their study, which was 380 patients and only included 22 high KDPI KT [19].

In a study by Tully et al, they reported one-year post transplantation outcomes in patients who received a high KDPI kidneys. They defined suboptimal outcomes as death-censored allograft failure or eGFR < 30 mL/min at the time point 1 y post-transplant, and allograft failure was considered to have occurred if recipients returned to dialysis, or recorded one-year eGFR < 10 mL/min. They have reported a suboptimal outcome of 44% in the recipients who received a kidney with a KDPI 96–100%, and 32% in the recipients who received a kidney with a KDPI 91–95, as expected [20]. Molinari et al. compared the eGFR, and rejection in different spectrum of KDPIs. The study mentioned that after the first year, patients in the KDPI above 85% group had a lower mean eGFR compared to the others, however, when the further analysis showed that there was not a significant difference in the speed of functional decline between the groups. (P = .06) Also, the slope of the eGFR curves were similar (P = 0.34). The rejection rate after the first year was 18.7% in KDPI < 20% group, 27% in the KDPI 21–85% group, and 32.3% in KDPI above 85% group (P = 0.03) [21]. Despite the statistical significance of the rejection rate, the clinical significance was not as important. There was a 6% difference between the KDPI 21–85% group and the KDPI above 85% group.

In our study, the KDPI 98–100% cohort, the donors’ age was slightly higher. In this older population, there may not be a need for longer allograft survival depending on the patient. Better outcomes were achieved for patients 35–64 years old receiving a kidney with KDPI 85–97%. As noted, most of the high KDPI kidneys are allocated to the patients who are 60 years old and above, based on the current kidney allocation system in the United States. For older patients, specifically those aged 65 to 69 years, the risks associated with waiting for a potentially better organ transplant offer are significantly increased. Essentially, for these patients, the time they can safely wait for a better organ is limited to 41 months before the risks of waiting outweigh the benefits. This emphasizes the urgency of not delaying a transplant in older patients [22]. Jay et al study also demonstrated the survival benefits of earlier kidney transplant with high KDPI kidneys in older than 60 years patients, instead of keeping them on the waiting list in both pre-emptive and non-preemptive KT. They found that the one- and 3-year patient survival rates were 96% and 90% for preKT in KDPI 0% to 85% recipients, 95% and 88% for preKT in KDPI greater than 85% recipients, 92% and 82% for non-preKT with KDPI 0% to 85% recipients, 91% and 79% for non-preKT in KDPI greater than 85% recipients, and 90% and 64% for waitlisted patients who were not transplanted during the study period (P < 0.01). One-year overall allograft survival reported was 93% for preKT with KDPI 0% to 85%, 90% for preKT with KDPI greater than 85%, 88% for non-preKT with KDPI 0–85%, and 84% for non-preKT with KDPI greater than 85%, respectively [23]. We also noticed a significant five-year allograft survival difference between the preKT and non-preKT patients in the KDPI 85–97% cohort in our study (67.3% vs. 53.3%, P < 0.001).

The study by Axelrod and colleagues focused on the economic aspects of high KDPI kidney transplantation and quality-adjusted life years (QALYs). They have found that during the 10-year study time, patients with high KDPI KT, has approximately gained 29% more survival, adjusted by different quality of life factors, compared to the ones who were on maintenance dialysis (5.20 vs. 4.03 QALYs). Also, transplantation with high KPDI kidneys, costs 12.2% less per QALY than the maintenance dialysis ($63,531 vs. $72,476 per QALY) [24].

Another study by Bamforth et al, in Canada demonstrated the same results. During a 10-year follow up, they found that the High KDPI kidney transplant cost per patients was $379,485, which it was $402,937 in patients on maintenance dialysis. Their model also found a higher QALY in high KPDI kidney recipients, compared to those were on maintenance dialysis (4.77 vs. 4.37). The mean cost-utility ratio in patients on maintenance dialysis and High KDPI kidneys were $92 205.42 and $79,556.67 per waitlisted patient. The difference in cost-utility ratios between the patients on maintenance dialysis and High KDPI kidneys was $12,648.75 per QALY [25].

Another study by Ellison et al has extensively compared the benefits and disadvantages of accepting a high KDPI kidney to staying on a waiting list to receive a lower KDPI kidney, using a Markov model, in terms of cost effectiveness and QALY. They discovered that the average cost savings per QALY was $154,600. As an example, their model found that there would be a $178,186 saving when a 65-year-old female with a BMI of 30, blood type A, White, panel reactive antibody of 25, renal failure from glomerulonephritis, no prior transplant, and with an estimated time on the waiting list of 36 months accept a kidney with a KDPI 91–100%.

It was found that 76.7% of patients received a kidney with a KDPI 81–90% and 59% in KDPI 91–100% would expect an increased QALY and 84.9% of patients received a kidney with a KDPI 81–90% and 69.7% in KDPI 91–100% would expect cost savings.

It was also demonstrated that 58.2%% of patients who received a kidney with a KDPI 81–90% and 38.8% in KDPI 91–100% would expect a survival benefit.

They have found that there was a 24.5% 5-year survival advantage by accepting a high-KDPI kidney for those with an estimated time to transplant ≥30 months, a PRA ≤ 89.5, age ≥ 55 years old, and having been previously transplanted [26].

These studies not only highlighted the importance of considering high KDPI kidneys, in terms of financial aspects, which can dramatically reduce the financial burden on the healthcare system, but also showed that how could the patients’ quality of life during the survival time improves, even with the High KDPI kidneys and possible suboptimal outcomes.

Delayed graft function (DGF) was found to be a strong predictor of allograft outcome. This adverse complication was found to significantly decrease the five-year allograft survival rate in both cohorts. DGF is largely attributed to either ischemia-reperfusion injury or innate immune activation. Risk factors for DGF include cold ischemic time, donor terminal creatinine, donor BMI, DCD and donor age however these are still under investigation [27].

In the KDPI 85–97% cohort, there was a higher number of kidneys allocated for transplantation from DCD donors. It was found that DCD donor classification did not have a significant effect on the outcome in our results. In a study from the UK, medium- and long- term outcome data are showing transplant outcomes from DCD donors are comparable to those of DBD donors. Renal function compared at both 5- and 10-years was similar between the study cohorts [28]. Increased utilization of these once marginalized kidneys helps to avoid the unnecessary kidney discard. Asian ethnicity demonstrated superior outcomes in both the 98–100% and 85–97 KDPI% cohort. Improved five-year allograft survival in the Asian population was demonstrated in Katznelson et al. too, where allograft survival rates for the Asian population was 66% compared to 61% for Hispanics and Whites, and 47% for Black patients. Superior allograft survival in the Asian population was speculated to be associated with higher prevalence of primary disease entities that are associated with improved long-term prognoses, similar to what we found during our study [29].

Chronic kidney transplantation rejection (CKTR) was found to be the main cause of allograft failure in both cohorts. CKTR maintains a complex pathogenesis and irreversibility at the time of diagnosis. Therefore, focus should be placed on the prevention and early management of rejection. The current mainstay prevention involves patient adherence to immunosuppressive medications. This involves regular medication level monitoring, patient education and communication between an interdisciplinary team [30]. New technology including biomarkers and therapeutic targets for CKTR are actively becoming identified and will hopefully be translated into clinical practice in the future [31].

Our study contributes to the growing body of evidence regarding the allocation of high KDPI kidneys, but we must acknowledge the limitations of our research. It should be noted that our data was pulled from the United Network for Organ Sharing (UNOS) database. The use of registration data can result in statistical analysis errors due to inaccurate or missing information. Although we tried to reduce and eliminate the effect of some confounders by matching the cohorts, using an unsupervised machine learning algorithm, there is still a chance that unmatched characteristics contribute some biases. Additionally, this study was a retrospective and longitudinal study, so there is a chance that controls were recruited according to convenience sampling, so bias in sampling is a possible factor. Future research must incorporate a more comprehensive assessment of these factors to gain a better understanding of the relationship between the high KDPI kidney allocation and KT outcomes.

In conclusion, this study was focused on how beneficial kidney transplantation using a high KDPI kidney is. There is no doubt that the long-term outcomes of high KDPI kidneys are suboptimal, compared to an idealized kidney transplant, but the question would be that are these kidneys good enough for the target group who receives these kidneys or not. Due to the structure of the current allocation system, and also the long list of patients on the waiting list, older patients and patients with specific genetic and immunization characteristics, usually remain on the waiting lists for a long period of time that they may not even survive till finding a good match. It has been shown that allocating high KDPI kidneys to these patients is beneficial, in terms of quality of life, financial aspects, and also patient survival, compared to keeping the patients on waiting list. By considering some of the recipients’ baseline characteristics, such as the age, history of maintenance dialysis, and the race, it is possible to have an optimal outcome, similar to what we have found in this study, in order to expand the donor pool and reduce the waiting time for the patients who are in the need of a kidney transplant.

## Abbreviations:

BMI: Body mass index
CIT: Cold ischemia time
DCD: Donation after cardiac death
CKTR: Chronic kidney transplantation rejection
DDKT: Deceased donor kidney transplant
DGF: Delayed graft function
ECD: Expanded criteria donor
ESRD: End stage renal disease
KDPI: Kidney Donor Profile Index
KT: Kidney transplantation
OPTN: Organ Procurement and Transplantation Network
SCD: Standard criteria donor
SRTR: Scientific Registry of Transplant Recipients
UNOS: United Network for Organ Sharing
